# Hepatoid adenocarcinoma of the lung: An analysis of the Surveillance, Epidemiology, and End Results (SEER) database

**DOI:** 10.1515/med-2021-0215

**Published:** 2021-01-22

**Authors:** Lei Lei, Liu Yang, Yang-yang Xu, Hua-fei Chen, Ping Zhan, Wen-xian Wang, Chun-wei Xu

**Affiliations:** Department of Chemotherapy, Institute of Cancer Research and Basic Medical Sciences of Chinese Academy of Sciences, Cancer Hospital of University of Chinese Academy of Sciences, Zhejiang Cancer Hospital, No.1 Banshan East Street, Gongshu District, Hangzhou, Zhejiang 310022, People’s Republic of China; Department of Medical, Shanghai Dunlu Biomedical Technology Co., Ltd., Shanghai 200032, People’s Republic of China; Department of Respiratory Medicine, Affiliated Jinling Hospital, Medical School of Nanjing University, 305 Zhongshan Road, Jiangsu, Nanjing 210002, People’s Republic of China; Department of Thoracic Disease Center, Zhejiang Rongjun Hospital, No. 589 Central West Road, Jiaxing, Zhejiang 314000, People’s Republic of China

**Keywords:** NSCLC, HAL, SEER, survival

## Abstract

Hepatoid adenocarcinoma of the lung (HAL) is a rare malignant tumor that is defined as a primary alpha-fetoprotein (AFP)-producing lung carcinoma. We aimed to identify prognostic factors associated with the survival of patients with HAL using data from the Surveillance, Epidemiology, and End Results (SEER) database. We collected data from patients diagnosed with HAL, adenocarcinoma (ADC), and squamous cell carcinoma (SCC) of the lung between 1975 and 2016 from the SEER database. The clinical features of patients with ADC and SCC of the lung were also analyzed. The clinical features of HALs were compared to ADCs and SCCs. A chi-square test was used to calculate the correlations between categorical variables, and a *t* test or Mann–Whitney *U* test was used for continuous variables. The Kaplan–Meier method and Cox regression analysis were used to identify the prognostic factors for the overall survival (OS) of HALs. Two-tailed *p* values < 0.05 were considered statistically significant. Sixty-five patients with HAL, 2,84,379 patients with ADC, and 1,86,494 with SCC were identified from the SEER database. Fewer males, advanced stages, and more chemotherapy-treated HALs were found. Compared to patients with SCC, patients with HAL were less likely to be male, more likely to be in an advanced stage, and more likely to receive chemotherapy (*p* < 0.05). The American Joint Committee on Cancer staging was the only prognostic factor for OS in patients with HAL, and stage IV was significantly different from other stages (hazard ratio = 0.045, 95% confidence interval: 0.005–0.398, *p* = 0.005). Males with HAL were more likely to receive radiotherapy compared to females with HAL (61.8 vs 31.5%, *p* = 0.034). Younger patients with HAL were more likely to receive chemotherapy (59.4 + 10.2 years vs 69 + 11.3 years, *p* = 0.001). The primary tumor size of HAL was associated with the location of the primary lesion (*p* = 0.012). No conventional antitumor therapies, including surgery, chemotherapy, and radiotherapy, were shown to have a significant survival benefit in patients with HAL (*p* > 0.05). This study showed that stage IV was the only prognostic factor for OS in HALs compared to other clinicopathologic factors. Conventional antitumor therapies failed to show survival benefit; thus, a more effective method by which to treat HAL is needed. Interestingly, the clinical features and the location of the primary lesion were shown to be associated with primary tumor size and treatment in patients with HAL, which have not been reported before.

## 1 Introduction

Hepatoid adenocarcinoma (ADC) is a rare but malignant tumor that commonly occurs in the stomach [1,2] and also been reported to originate from the pancreas [3], bladder [4], and ovary [5]. The liver-like ADC originating from the lung is referred to as hepatoid adenocarcinoma of the lung (HAL), which has a low incidence (2.3%) among all hepatoid ADCs and a poor prognosis [6]. The median overall survival (OS) of patients with HAL is only 5 months and the 1- and 3-year survival rates are 35 and 14%, respectively [7]. Given the small number of case series and short follow-up interval after surgery, the inconsistent description of characteristics and prognosis of patients with HAL are inevitable.

HAL generally has no specific clinical symptoms and metastatic site predilection, but computed tomography (CT) images may support the diagnosis [8]. The final diagnosis should be confirmed by histologic and immunohistochemical (IHC) methods, which can discriminate HAL from hepatocellular carcinoma [9]. Compared with common types of lung cancer, the poor prognosis of HAL has been reported in several studies [10,11,12]. The prognostic biomarker for HAL remains to be established although an elevated serum α-fetoprotein (AFP) level and positive AFP expression on IHC analysis have been reported as poor prognostic markers [13]. Moreover, there is no standard treatment for HAL. In general, localized HAL surgical be resected, and platinum-based chemotherapy and/or radiation treatment is recommended for patients with advanced or metastatic disease [14]. Nevertheless, the OS is still extremely poor in most patients in late clinical stage HAL [9].

Previous studies have mainly focused on the description of clinical features and the outcome of patients with HAL in case series. Recently, Ayub et al. [7] described a patient with HAL who had an aggressive course of disease and reviewed 41 cases from the SEER database from 1988 to 2014. A large database analysis is still lacking due to the extremely low incidence of this type of lung cancer.

In this study, we reviewed 65 cases of HAL in the same database from 1975 to 2016 and compared the clinicopathologic characteristics of ADC and squamous cell carcinoma (SCC) of the lung to expand the knowledge base pertaining to the clinical features, treatment, and OS-related factors in patients with HAL.

## 2 Material and methods

### 2.1 Patient cohort

The Surveillance, Epidemiology, and End Results (SEER) is a free public cancer database. SEER collects data from 18 geographic registries, representing approximately 30% of the US population. We applied for an account to access data and to determine frequency rates. The inclusion criteria for this study included patients with a diagnosis of hepatoid ADC between 1975 and 2016, and a histologic type (code 8576) according to the third edition of the International Classification of Diseases for Oncology (ICD-O-3). The primary site was selected as C34, and the location of the tumor was limited to the lung. There were 65 patients with HAC, 2,84,379 patients with ADC, and 1,86,494 patients with SCC identified from the SEER database.


**Ethical approval and consent to participate:** The clinical data used were from the SEER database, which is a public research resource; patient consent and ethical approval for the study were not required.

### 2.2 Statistical analysis

The incidence and clinical trends for HAC were analyzed using SEER*Stat version 8.3.6 (National Cancer Institute, Bethesda, MD, USA). The incidence was age adjusted to the 2000 US standard population. Annual percentage changes were calculated using the weighted least square method. The clinical factors were analyzed using descriptive statistics, and the chi-square test was used to calculate correlations between categorical variables. Kaplan–Meier curves were generated to assess disease-specific survival, and the differences between groups were compared using log-rank analysis. Cox proportional hazard regression was performed on demographic, clinical, and treatment factors to estimate the survival differences. Cox regression analysis was used for factors that had statistical significance based on univariate analysis. We processed and analyzed the data using statistical software R (version 3.34; http://www.r-project.org). A *p* < 0.05 indicated statistical significance.

## 3 Results

### 3.1 Clinicopathologic characteristics

A total of 65 HALs were identified from the database, including 31 male and 34 female patients. The median age at the time of HAL diagnosis was 64.11 ± 11.704 years, which is similar to the median age at the time of ADC and SCC diagnoses during the same period. There were fewer male patients with HAL than with SCC (47.7 vs 64.8%, *p* < 0.05). The advanced stages (T3-4 and M1) were more prevalent in HALs than in SCCs (both *p*s < 0.05). In addition, patients with HAL were more likely to receive chemotherapy than patients with SCC (50.8 vs 31.8%, *p* < 0.001). Race (white vs not white), lymph node metastases, undergoing surgery, and receiving radiotherapy were not significantly different between patients with HAL and patients with both ADC and SCC (Table 1).

**Table 1:** Comparison between HAC, ADC, and SCC

Variables		HAC (*n* = 65)	ADC (*n* = 2,84,379)	SCC (*n* = 1,86,494)
Age		64.11 ± 11.704	66.39 ± 11.47	68.93 ± 9.88
Sex (%)	Male	31 (47.7)	91,755 (48.7)	68,381 (64.8)
	Female	34 (52.3)	96,830 (51.3)	37,220 (35.2)
Race (%)	White	55 (84.6)	1,52,422 (80.8)	87,126 (82.5)
	Not white	10 (15.4)	36,163 (19.2)	18,475 (17.5)
T stage (%)	NA	36 (55.3)	2,10,280 (43.9)	1,46,471 (78.5)
	T0	1 (1.5)	629 (0.22)	126 (0.1)
	T1	3 (4.6)	17,720 (6.2)	7,017 (3.8)
	T2	5 (7.7)	19,942 (7.0)	13,406 (7.2)
	T3	10 (15.4)	15,583 (5.5)	9,716 (5.2)
	T4	10 (15.4)	17,705 (6.2)	9,758 (5.2)
N stage (%)	NA	31 (47.7)	2,05,668 (72.3)	1,45,153 (77.8)
	N negative	16 (24.6)	31,396 (11.0)	17,710 (9.5)
	N positive	18 (27.7)	47,315 (16.6)	23,631 (12.7)
M stage (%)	NA	29 (44.6)	2,00,134 (70.4)	1,43,039 (76.7)
	M0	12 (18.5)	35,650 (12.5)	27,957 (15.0)
	M1	24 (36.9)	48,595 (17.1)	15,498 (8.3)
Surgery (%)	NA	56 (86.2)	5,217 (1.8)	1,28,918 (69.1)
	No	1 (1.5)	2,01,535 (70.9)	5,429 (2.9)
	Yes	8 (12.3)	77,627 (27.3)	52,147 (27.9)
Radiation (%)	No/unknown	32 (49.2)	1,14,066 (40.1)	91,998 (49.3)
	Yes	33 (50.8)	1,70,313 (59.9)	94,496 (50.7)
Chemotherapy (%)	No/unknown	32 (49.2)	1,71,733 (60.3)	1,28,330 (68.8)
	Yes	33 (50.8)	1,12,646 (39.6)	58,164 (31.8)

### 3.2 Survival analysis in patients with HAL

**Figure 1:**
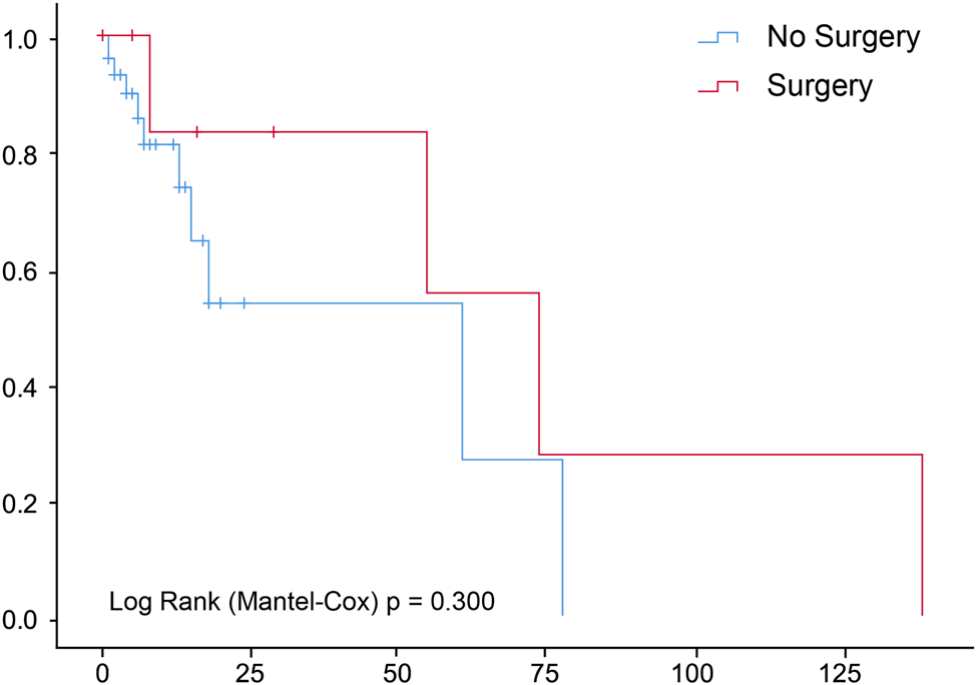
Surgery does not impact survival of patients with hepatoid adenocarcinoma of the lung.

**Figure 2:**
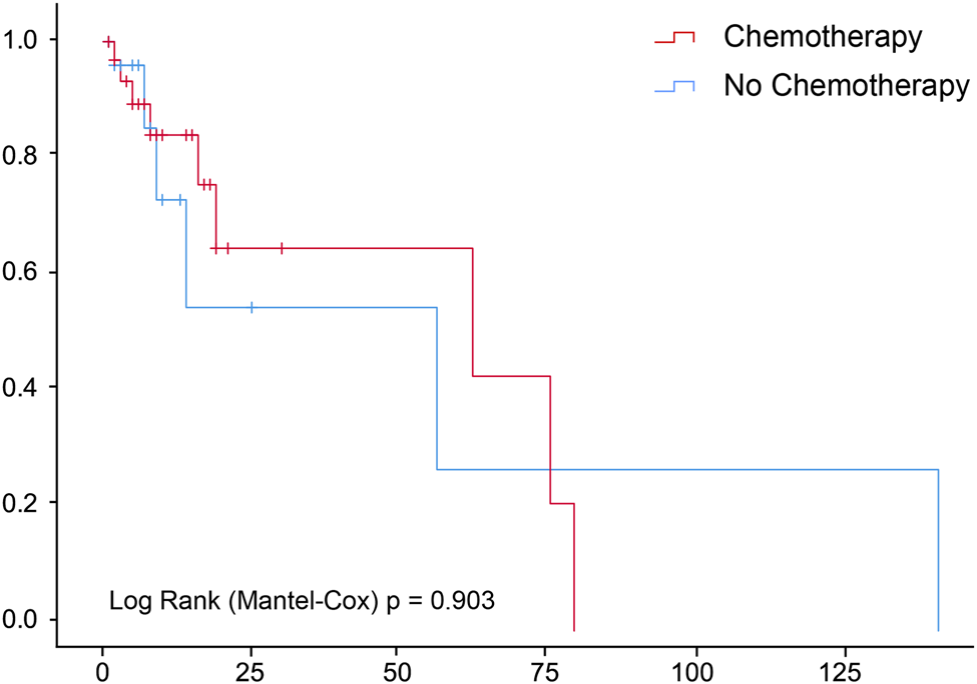
Chemotherapy does not impact survival of patients with hepatoid adenocarcinoma of the lung.

**Figure 3:**
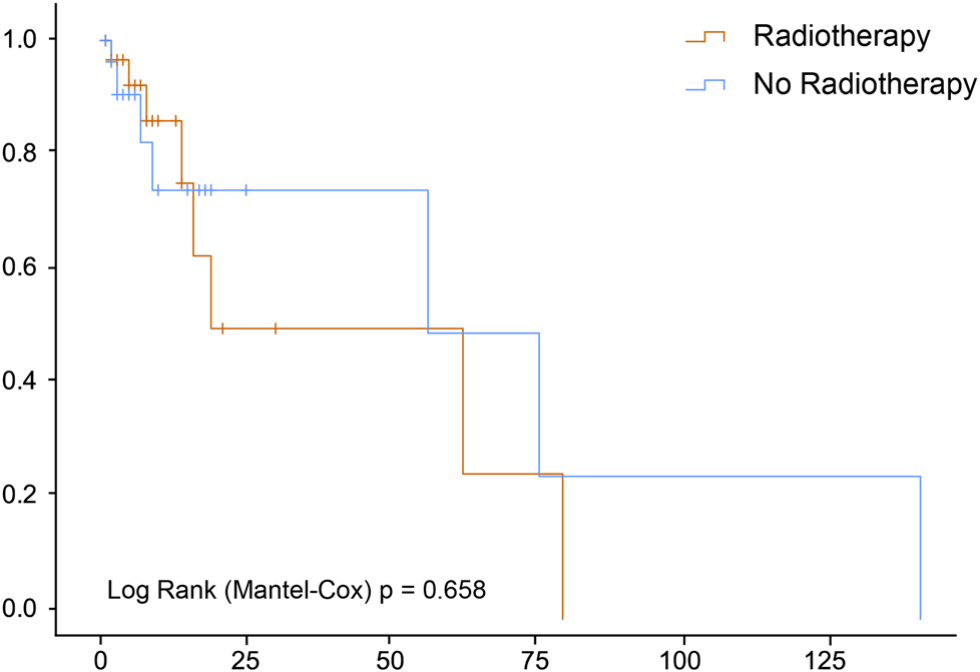
Radiotherapy does not impact survival of patients with hepatoid adenocarcinoma of the lung.

The median OS of the 65 patients with HAL was 5 months (95% confidence interval [CI], 2.55–7.45); the 3- and 5-year survival rates were 7.7 and 6.2%, respectively. A total of 50 patients died during the follow-up period, of which 48 were cancer-specific and 2 were due to other causes (chronic obstructive pulmonary and heart diseases). Multivariant analysis of prognostic factors for OS prediction in patients with HAL included age (hazard ratio [HR], 1.03; 95% CI, 1.01–1.04), chemotherapy (HR, 0.51; 95% CI, 0.36–0.73), and surgery (HR, 0.53; 95% CI, 0.36–0.77), all of which were associated with cancer-specific survival. The American Joint Committee on Cancer (AJCC) staging was the only prognostic factor for OS in patients with HAL; stage IV HAL was significantly different from other stages (HR, 0.045; 95% CI, 0.005–0.398, *p* = 0.005; Table 2). No conventional antitumor therapies, including surgery, chemotherapy, and radiotherapy, were shown to have a significant survival benefit in patients with HAL (*p* > 0.05; Figures 1–3).

**Table 2:** AJCC stage is the only hazard factor in the Cox proportional hazards model

AJCC stage	*B*	HR	95% CI	*p*
NA	—	—	—	—
IA	−2.617	0.073	0.004–1.233	0.070
IB	−24.503	0.000	0.000–1.500 × 10^170^	0.908
IIB	−14.485	0.000	0.000–2.897 × 10^259^	0.963
IIIA	−13.218	0.000	0.000–8.243 × 10^119^	0.929
IIIB	−24.157	0.000	0.000–2.125 × 10^170^	0.909
IV	−3.091	0.045	0.005–0.398	0.005

### 3.3 Correlation analysis of clinical features in patients with HAL

Male patients with HAL were more likely to receive radiotherapy compared with female patients with HAL (61.8 vs 31.5%, *p* = 0.034). Patients with HAL treated by chemotherapy were more likely to be younger (59.4 + 10.2 years vs 69 + 11.3 years, *p* = 0.001). Of the 51 patients with HAL, 30 (58.8%) had primary tumors located in the upper lobes of the lungs, with a median tumor diameter of 173.2 ± 306.2 mm. Ten (19.6%) of 51 HALs with the largest median tumor diameter (626.6 ± 481.9 mm) were located in the lungs with no specific anatomic location. Of 51 HALs, 9 (17.6%) were in the lower lobes of the lungs, with a median tumor diameter of 151.8 ± 318.7 mm. One patient had a tumor in the main bronchus (60 mm in diameter) and a patient had a tumor in the middle lobe (46 mm in diameter). HAL size was associated with the location of the primary lesion (*p* = 0.012).

## 4 Discussion

In this study, we analyzed 65 patients with HAL from the SEER database between 1975 and 2016 and found that the clinical stage was the only OS-related factor. Surgery, chemotherapy, and radiotherapy had no significant survival benefit in patients with HAL. Compared with the common types of lung cancer, HALs tended to have closer clinicopathologic characteristics to ADCs than SCCs. Of note, we also identified interactions between gender and radiotherapy, age at diagnosis and chemotherapy, and the primary site and tumor size.

Clinically, HALs are considered to be male dominant with an abnormal AFP level in the serum or expressed on tumor tissue [10,15]. Male patients with HAL have been reported to be ninefold more frequent than female patients with HAL [8]; however, the percentages of males and females with HAL in our study (47.7 vs 52.3%) were closer to the results from a recent study [7]. Furthermore, the prevalence of male patients with SCC was significantly higher than male patients with HAL (64.8 vs 47.7%; *p* < 0.05). An abnormal AFP level has been associated with poor outcome in patients with HAL and other hepatoid ADCs [10,14,16]; however, the AFP elevation in serum and higher AFP expression in tumor tissues are not necessary criteria for HAL diagnosis [15].

Of note, we further explored the association among those clinical features of HALs which have not been previously reported. We showed that greater than one half (30/51) of primary HALs was located in the upper lobes of the lungs, which is consistent with previous data [7,15,17,18]. The mean size of the primary tumor has been reported to be approximately 7 cm (range, 1–20 cm) [11,18]. At the time of initial diagnosis, lymph node involvement is more common in male patients with HAL than female patients with HAL [8]. In this study, we also found that the number of male patients who received radiotherapy was twice that of female patients. Currently, mediastinal tissue sampling before surgical resection of a HAL is not recommended [7].

In this study, the clinical stage was the only prognostic factor for OS in patients with HAL. Although promising prognostic indicators for HALs are still lacking, the clinical stage is the most likely OS-related factor based on several studies. Patients with stages I–II HAL could have a disease-free survival of up to 7 years after surgery and adjuvant therapy [19,20,21]. These findings also suggest that curative resection and reasonable adjuvant chemotherapy and/or radiotherapy may influence the outcome of HALs; however, our findings did not show a statistically significant OS benefit from any of the abovementioned therapies. A better understanding of the biology of this rare but aggressive disease could facilitate the identification of effective therapeutic targets and drugs in the future.

Currently no standard guidelines are available for the treatment of HALs; however, surgical resection for patients in an early stage and palliative chemotherapy with platinum and radiotherapy for patients in a late stage are recommended. Although we did not present an analysis of specific chemotherapies for HALs due to a lack of such data in the SEER database, the lack of OS benefit by all of the conventional therapies in our study is noteworthy. Fortunately, encouraging data by new therapies have recently been reported. Specifically, a patient with HAL stage IV had a good response and tolerated targeted therapy with sorafenib and platinum-based chemotherapy; the median OS was 11 months [22]. A patient with an HAL stage IV has been reported to respond surprisingly well to anti-PD-L1 durvalumab therapy, despite all negative test results for EGFR, KRAS, ALK, ROS1, and PD-L1 [23].

Several limitations of this study should be mentioned. First, it was a retrospective public database analysis and bias of case selection could not be excluded. As far as we know, however, this is the first study of HALs by way of comparison to a large number of common NSCLC types. Second, information about potential prognostic factors, including the serum AFP level, smoking status, CT findings, and genetic mutations, are not included in the SEER database. In our analysis, we included all available clinicopathologic factors for HALs from the SEER database. Finally, due to the limited number of HALs, the comparison results of HALs with ADCs and SCCs should not be applied in practice due to the potential statistical bias. Furthermore, we did not perform a subgroup analysis of treatments by different stages for survival benefit. A corollary study with a larger sample size of patients with HAL should be conducted to verify our conclusions.

## 5 Conclusion

In summary, we found that HALs are an aggressive and highly heterogeneous type of lung cancer, thus earlier diagnosis and better treatment are needed. HAL stage IV was the only negative prognostic factor for OS compared with other clinical stages. Interestingly, gender, age at the time of diagnosis, and location of the primary lesion could influence treatment and tumor size of HALs, which have not been previously reported.
